# Targeting the HPV E6–p53 degradation axis via computational and *in vitro* identification of a repurposed small-molecule candidate

**DOI:** 10.3389/fonc.2026.1913599

**Published:** 2026-07-28

**Authors:** Shamanth D. Harishkumar, Shuaib Pasha, Rosemary Edwin, Sahana Madashetty, Bhargav Shreevatsa, Shiva Prasad Kollur, Chandan Shivamallu, Chandan Dharmashekar

**Affiliations:** 1 Department of Biotechnology and Bioinformatics, School of Life Sciences, JSS Academy of Higher Education and Research, Mysuru, Karnataka, India; 2 Department of Microbiology, School of Life Sciences, JSS Academy of Higher Education and Research, Mysuru, Karnataka, India; 3 Department of Grain Science and Technology, CSIR - Central Food Technological Research Institute, Mysuru, Karnataka, India; 4 School of Physical Sciences, Amrita Vishwa Vidyapeetham, Mysuru, Karnataka, India

**Keywords:** adenosine, cervical cancer, cytotoxicity, drug repurposing, molecular dynamics simulation, network pharmacology

## Abstract

**Background:**

High-risk HPV infection initiates cervical cancer through E6-mediated degradation of the p53 tumor suppressor protein via the ubiquitin-proteasome system, leading to apoptotic failure and genomic instability. No FDA-approved therapies currently target HPV E6, representing a significant unmet clinical need. This study aimed to identify repurposable compounds predicted to engage the p53 interface disrupted by E6-mediated proteasomal degradation, as a starting point for future p53-stabilisation studies.

**Methods:**

An integrated computational and experimental repurposing strategy was applied, encompassing network pharmacology, molecular docking, density functional theory (DFT), in silico ADMET profiling, and 500 ns molecular dynamics simulations. Network analysis was used to identify the primary hub protein targeted by HPV E6. Candidate compounds were screened for binding affinity against p53 chains C and D within the HPV16-associated degradation complex (PDB: 4XR8). *In vitro* validation was conducted by antioxidant, anti-inflammatory, anti-angiogenic, and cytotoxicity assay was assessed in SiHa (HPV16-positive) cells.

**Results:**

Network analysis confirmed TP53 as the principal hub disrupted by the HPV E6 protein. Among all screened compounds, adenosine demonstrated the highest binding affinities against p53 chains C and D (−6.318 kcal/mol and −7.104 kcal/mol, respectively), suggesting a stabilizing interaction at the p53 interface. DFT calculations revealed moderate electronic stability, with a HOMO–LUMO frontier orbital energy gap of 5.399 eV. ADMET profiling indicated an acceptable pharmacokinetic profile, and molecular dynamics simulations showed lower RMSD and RMSF fluctuations compared to doxorubicin over 500 ns. *In vitro* assays demonstrated dose-dependent cytotoxicity against SiHa cells (IC_50_ = 20.6 µg/mL), alongside antioxidant, anti-inflammatory, and anti-angiogenic activities.

**Conclusion:**

Adenosine represents a promising drug repurposing candidate for further investigation as a modulator of the HPV E6–p53 interaction, exhibiting favorable computational and preliminary *in vitro* profiles. However, adenosine’s short plasma half-life and complex receptor-mediated effects present major translational barriers, and direct biochemical evidence of p53 stabilization is still required to confirm this mechanism. Further mechanistic and translational studies are warranted to establish its therapeutic efficacy and clinical applicability.

## 1 Introduction

Cervical malignancy is a neoplasm of the cervical epithelial tissue; this process predominantly occurs in the transition zone, at the interface between ectocervical squamous epithelium and endocervical columnar epithelium (1, 2). The most frequently encountered histological type, squamous cell carcinoma, makes up the majority (70–80%) of cervical cancer, with adenocarcinoma representing 10–20% of cases (1, 3). Other less frequently encountered types of cervical cancer include small cell neuroendocrine carcinoma, adenosquamous carcinoma, and additional rare histological subtypes (3).

Squamous cell carcinoma arises from a pre-existing lesion (4, 5). The adenocarcinoma originates from the glandular epithelium in the endocervical region, which has received very little attention until recently, based on the overwhelming quantity of published literature with respect to the general experience and from the fact that traditional cytological screening has not adequately separated the glandular epithelium from other types of squamous epithelium for the purpose of identifying abnormalities (4). Cervical cancer is pathologically characterized by the uncontrolled proliferation of epithelial cells: squamous carcinoma is usually accompanied by nests and sheets of discordant or keratinizing epithelial cells; for adenocarcinoma the glandular elements are often arranged in a haphazard, non-malignant manner with a great deal of mitotic activity (1, 3).

Cervical cancer is caused by lingering infections of high-risk HPV types (notably 16 and 18) (5). It is the fourth most common cancer in females globally, with approximately 604,000 new cervical cancer cases and 342,000 deaths worldwide (6, 7). HPV E6 oncogene hijacks the host ubiquitin–proteasome system to degrade p53 (8). Scheffner et al. demonstrated that HPV-16/18 E6 recruits wild-type p53 and E6AP to mediate ATP-dependent ubiquitination and proteasomal degradation of p53 (8, 9). In inducing the selectivity of p53 degradation, E6 relieves normal cellular stress mechanisms meant to lead to p53-mediated cell-cycle arrest, apoptosis, or DNA repair (9, 10). Thus, with inactive p53, HPV-infected cells acquire the ability to divide uncontrollably under genotoxic cellular stress and permit accumulation of mutations and malignant progression (9, 11). In addition to p53, E6 can also promote degradation of other cellular targets, such as PDZ-domain proteins that promote altered cell polarity; however, p53 inactivation remains a primary driver of E6-dependent transformation (11, 12). Therefore, persistent expression of E6 is necessary for the survival of cancer cells, representing a rational viral target for therapeutic intervention.

Despite improvements in screening and vaccination, many women continue to present with invasive or recurrent cervical cancer. Current standard treatments include surgery, radiation, and cytotoxic chemotherapy; however, the long-term prognosis with advanced disease is poor. Platinum-based regimens (cisplatin) combined with radiotherapy remain the standard of care, with frequent recurrences even with the best therapy (13). Bevacizumab was approved for use with chemotherapy for cervical cancer after demonstrating only a minimal median survival advantage (~3.5 months), with significant toxicity (14). No curative treatment currently exists for recurrent or metastatic cervical cancer. Other targeted agents against VEGF, EGFR, and other relevant pathways have failed to show sustainability. More recently, immune checkpoint inhibitors such as pembrolizumab have been approved for PD-L1-positive tumors (15). In the recurrent or metastatic setting following first-line therapy, single-agent chemotherapies demonstrate only markedly limited clinical effect (16). Crucially, none of the FDA-approved therapies target the viral aetiology — there is not a single drug that specifically inhibits HPV E6 or reactivates tumor suppressors suppressed by viral infection (11, 12). This critical unmet medical need underscores the importance of E6-targeting strategies that would directly address the fundamental mechanism of HPV-mediated malignancy.

Developing a new E6 inhibitor from scratch usually requires at least a decade and costs billions of dollars (17, 18). Drug repurposing identifies alternative therapeutic applications of approved drugs, offering a fast track for drug development; pharmacokinetics and safety have already been demonstrated for approved compounds, which saves enormous time and expense (17, 18). Repurposed drugs are cheaper and have a known toxicity profile (18). Repurposed drugs have a long history in oncology: thalidomide was repurposed to treat multiple myeloma, and arsenic trioxide was repurposed to treat acute promyelocytic leukaemia (17). Preliminary computational studies have explored repurposing approved drugs for cervical cancer, including docking-based screens identifying antiviral drugs valganciclovir and cytarabine as candidates that could bind to HPV E6 (19). The therapeutic strategy of targeting a viral oncoprotein like HPV E6 is potentially highly specific, since E6 has no homologue in normal human cells, meaning inhibitors should uniquely affect HPV-infected tumor cells while leaving healthy tissue unaffected (11, 12). Theoretically, blocking E6 may permit endogenous p53 levels to recover, enabling malignant cell apoptosis or senescence (10, 20). A number of recent proof-of-concept studies illustrate the potential value of E6-directed therapy; for example, a designed PROTAC molecule leading to degradation of HPV16 E6 inhibited tumor growth in a mouse model of HPV-positive tumors (21, 22). Therefore, we adopted both *in silico* and *in vitro* strategies to systematically repurpose FDA-approved compounds targeting the p53 interface disrupted by HPV E6, with the goal of identifying agents that may stabilise p53 and counteract E6-mediated oncogenesis. Beyond the direct E6-p53 axis, chronic oxidative stress, sustained inflammation, and pathological angiogenesis are separately established contributors to HPV-driven cervical carcinogenesis (23). A repurposed candidate offering combined antioxidant, anti-inflammatory, and anti-angiogenic activity alongside engagement of the p53 pathway would therefore carry added translational value even where these effects act independently of E6-mediated p53 degradation. FDA-approved drug compounds were sourced from the Pharmacodia Global Database for screening against the target protein (24).

## 2 Materials and methods

### 2.1 Target protein functional analysis

The Gene Expression Omnibus (GEO) database (25, 26) (accessed on 20 January 2026) was used to source and analyze two datasets (GSE100681 and GSE58841) derived from HPV E6/E7-expressing keratinocyte models; while these datasets are relevant to HPV-driven oncogenic transformation, they do not represent primary cervical tumor tissue and this distinction should be considered when interpreting the biological significance of identified hub genes. Gene expression profiling identified DEGs using an adjusted p-value cutoff of 0.05 and log-fold change (logFC) ≥ 2.

Two groups of common DEGs were identified for both datasets using a Venn diagram analysis. The protein–protein interaction (PPI) network was built using the STRING database (27) (accessed on 24 January 2026) with a very high confidence level (0.9) and imported into Cytoscape (28) (accessed on 24 January 2026) for topology analysis. The Network Analyzer plug-in was used to compute centrality using degree (≥15), closeness (≥0.45), and betweenness (≥0.10) centrality parameters. Multiple algorithms for gene ranking of core hub genes were used, and those consistently ranked high were considered significant hubs. Hub genes were then analyzed for disease ontology and cell-type enrichment using the Metascape tool (29) (accessed on 25 January 2026).

### 2.2 *In silico* molecular docking

Molecular docking was performed using Schrödinger Suite (Maestro) (30) to evaluate ligand–protein binding interactions. Protein structure coordinates were downloaded from the Protein Data Bank and prepared using the Schrödinger Protein Preparation Wizard, which includes adding missing side chains, assigning bond orders, optimizing hydrogen bonding, and energy minimization using the OPLS4 force field (31). Ligand structures were processed using LigPrep to assign appropriate ionization states at physiological pH. A grid-based receptor model was established centered on the active site. Docking was conducted using Glide (SP) and binding affinities analyzed using GlideScore and interaction mapping.

### 2.3 DFT calculations

Density functional theory (DFT) calculations were conducted using the Jaguar module of Schrödinger Suite (32) to investigate electronic properties of ligands. Ligand geometries were optimized using the B3LYP functional with appropriate basis sets. Key quantum parameters (HOMO–LUMO gap, molecular electrostatic potential, dipole moment) were computed to assess electronic characteristics, reactivity profiles, and protein interaction capacity.

### 2.4 ADMET study

ADMET profiles were assessed in silico to predict pharmacokinetic and toxicity profiles of selected ligands using SwissADME (33), pkCSM (34), and ADMETlab3.0 (35). Parameters evaluated include absorption, distribution, intestinal absorption, cytochrome P450 metabolism, excretion, and toxicity prediction. These predictions provide insights for drug-likeness, safety, and bioavailability applicable to candidate selection for further docking and experimental validation.

### 2.5 Molecular dynamics-based stability analysis of protein–ligand complex

Molecular dynamics (MD) simulations were conducted to investigate the conformational stability and dynamic behavior of protein–ligand complexes (36). The Desmond module of Schrödinger Suite was used, employing the OPLS4 force field. The protein–ligand complex was immersed in a TIP3P water model (37), surrounded by an orthorhombic boundary box, neutralized by counter-ions, and energy minimized. Equilibration was performed under NPT ensemble conditions. Production runs of 500 ns were performed, and trajectories were analyzed for RMSD and RMSF.

### 2.6 Free radical scavenging activity using DPPH

The DPPH radical scavenging assay quantifies antioxidant potential (38) at five different sample concentrations (3.125–100 µg/mL). A volume of 3 mL of DPPH was added to each sample and incubated for 30 minutes, then absorbance was recorded at 517 nm. Ascorbic acid (vitamin C) was used as the standard.

### 2.7 FRAP-based antioxidant activity assessment

The reduction of Fe^3+^ to F^2+^ generating a chromogenic product was quantified spectrophotometrically at 700 nm to evaluate antioxidant activity (39). The reaction components were phosphate buffer, diluted sample (1:10), and deionized water. Potassium ferricyanide (2.5 mL of 10%) was added to initiate the reaction, followed by incubation in a water bath at 50 °C for 20 minutes. Trichloroacetic acid (5 mL) was then added to terminate the reaction. The reaction mixture was centrifuged, and the supernatant was collected. Distilled water and iron (III) chloride were added to the supernatant, and absorbance was measured at 700 nm.

### 2.8 Anti-inflammatory assay

Lipoxygenase inhibitory potential of the test samples was evaluated using a protocol adapted from (40), using 5-lipoxygenase (5-LOX) as enzyme source and linoleic acid as substrate. The reaction medium consisted of 1 mL sodium borate buffer (0.1 mM, pH 8.8), 10 µL lipoxygenase enzyme (final concentration 8000 U/mL), and 1 mL of test sample at concentrations of 15.62 to 1000 µg/mL. After incubation for 5 minutes at 30 ± 2 °C, the reaction was initiated by addition of 10 µL linoleic acid (10 mM). Lipoxygenase inhibition was quantified by measuring absorbance at 234 nm using UV-visible spectrophotometry.

### 2.9 MTT-based cytotoxicity assay

The MTT assay (3-(4,5-dimethylthiazol-2-yl)-2,5-diphenyltetrazolium bromide) was used to assess cytotoxic effects (41). Cells were plated and allowed to settle for 12 hours to allow strong adhesion, then treated with the test compound at concentrations of 10-320 µg/mL. After 24 hours of treatment, 20 µL of MTT solution (5 mg/mL) was added to each well and incubated for 3 hours at 37 °C under CO2 incubator conditions. The medium was carefully aspirated and replaced with 100 µL of dimethyl sulfoxide (DMSO) to disperse the purple formazan crystals. Optical density was measured at 570 nm using a microplate reader.

### 2.10 CAM assay

Anti-angiogenic potential was assessed using the chorioallantoic membrane (CAM) assay (42, 43). Fertilized chicken eggs were maintained at 37 ± 1 °C and 60% relative humidity for 7 days. Small windows were made through the shell for exposure of the CAM. Test sample was applied on sterile discs at predetermined concentrations and placed gently on the CAM surface. After 24 hours of incubation, embryos were assessed under a stereomicroscope for vascular density, branching, and inhibition of new vessel formation.

## 3 Results:

### 3.1 Target protein functional analysis

**Figure 1:**
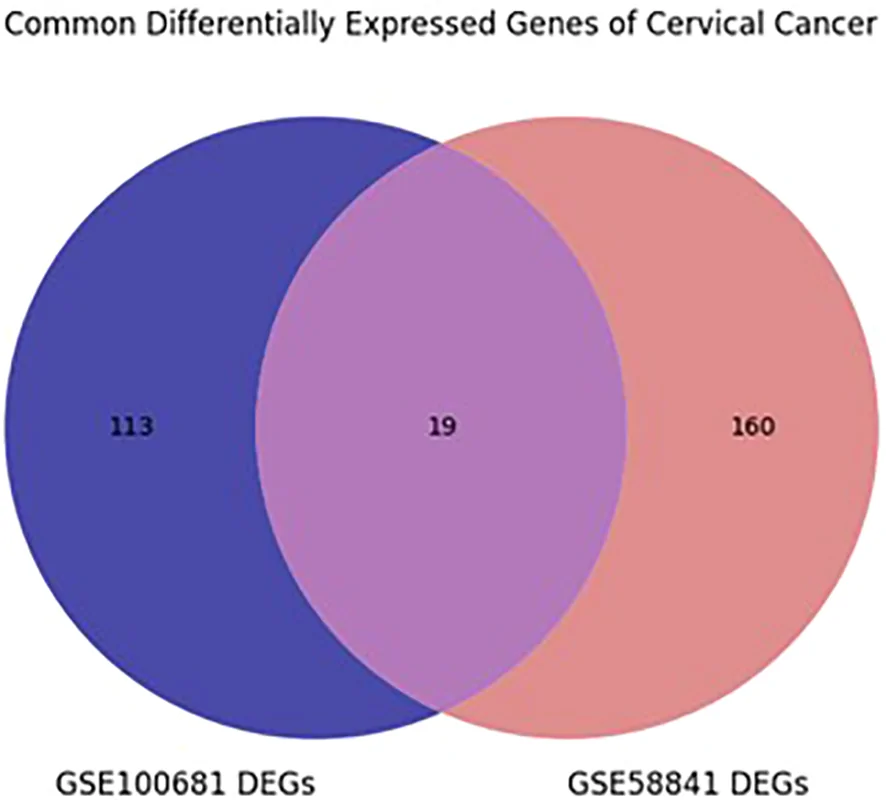
Venn diagram of differentially expressed genes between GSE100681 and GSE58841 datasets.

**Figure 2:**
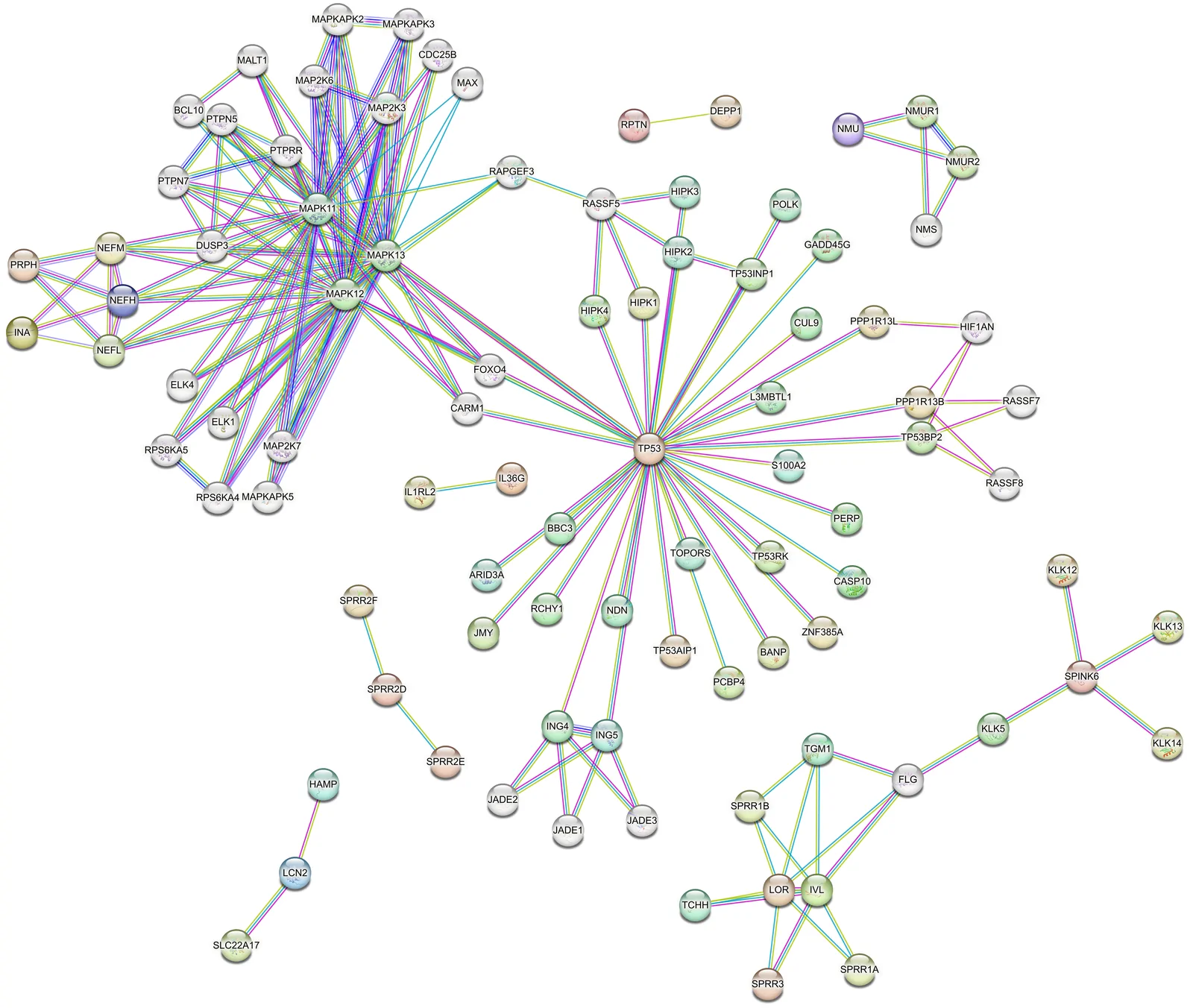
PPI network analysis of genes exhibiting differential expression across experimental conditions.

**Figure 3:**
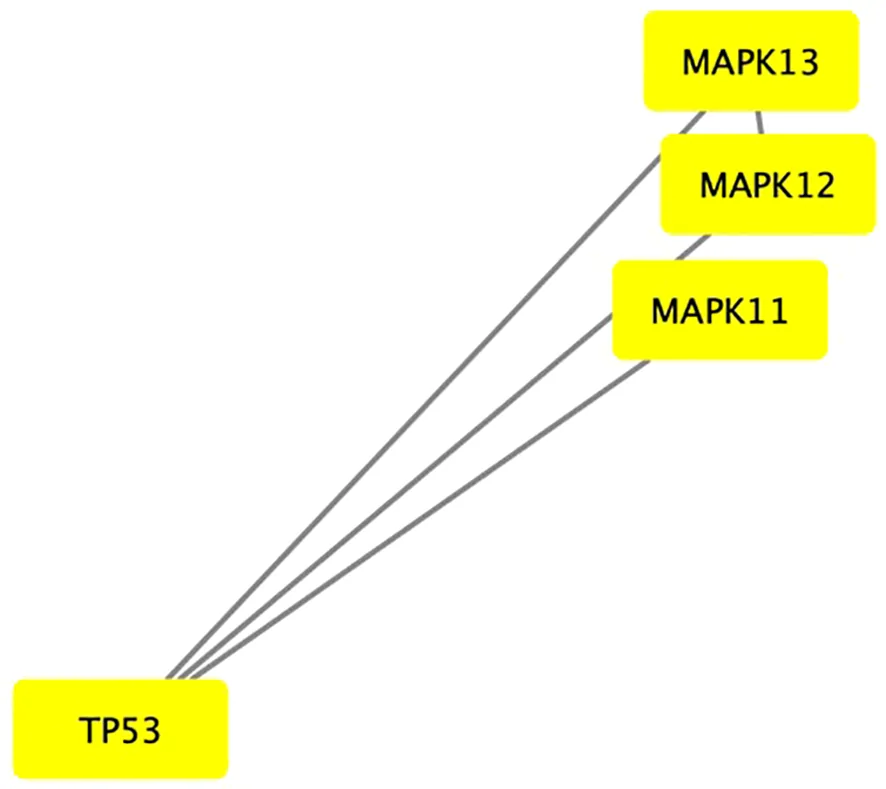
Network centrality-based filtering (degree, closeness, and betweenness) identifying key hub genes.

**Figure 4:**
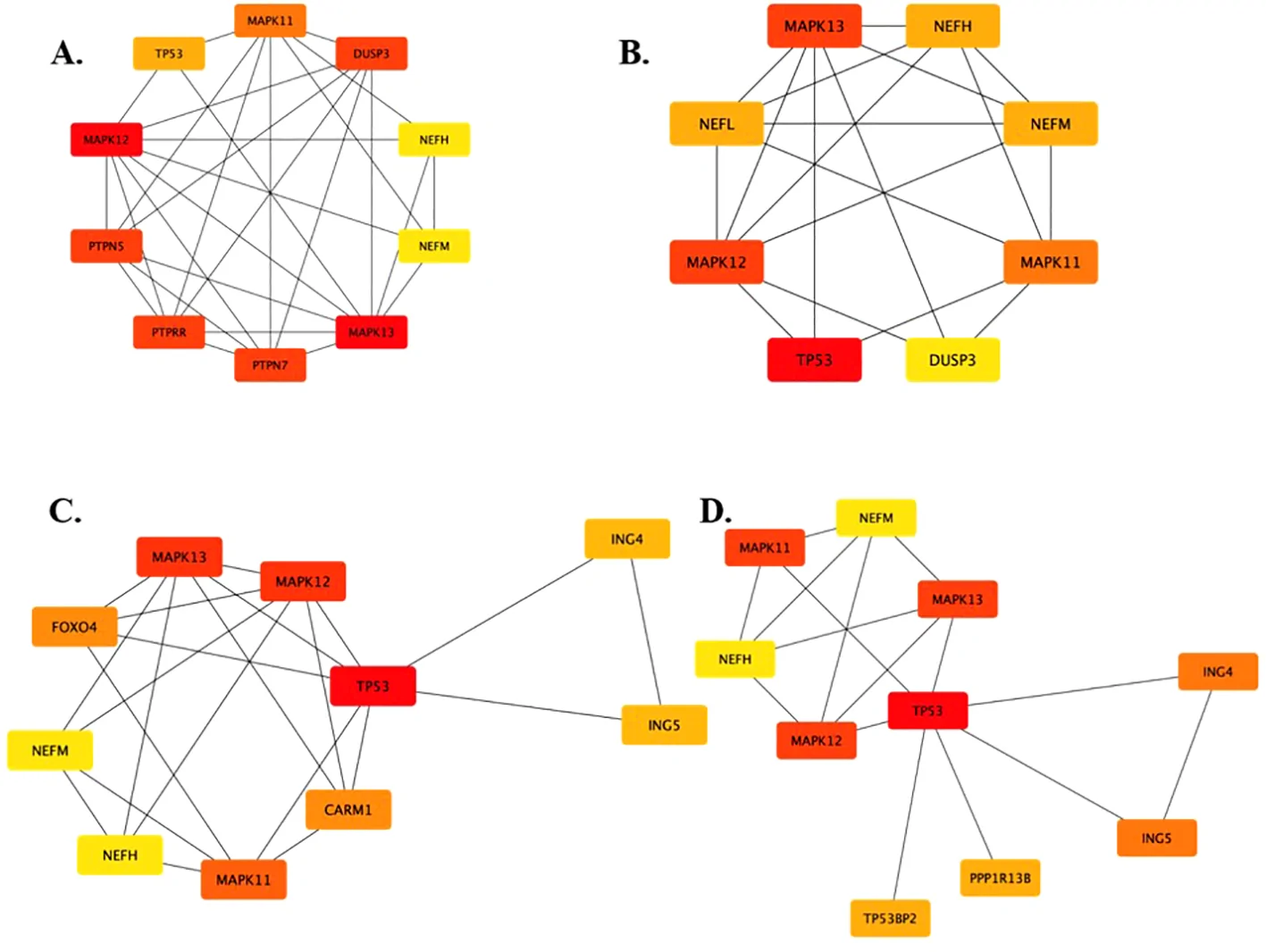
Comparative visualisation of hub gene ranking across **(A)** MCC, **(B)** degree, **(C)** closeness, and **(D)** betweenness centrality algorithms, highlighting dominant nodes.

**Figure 5:**
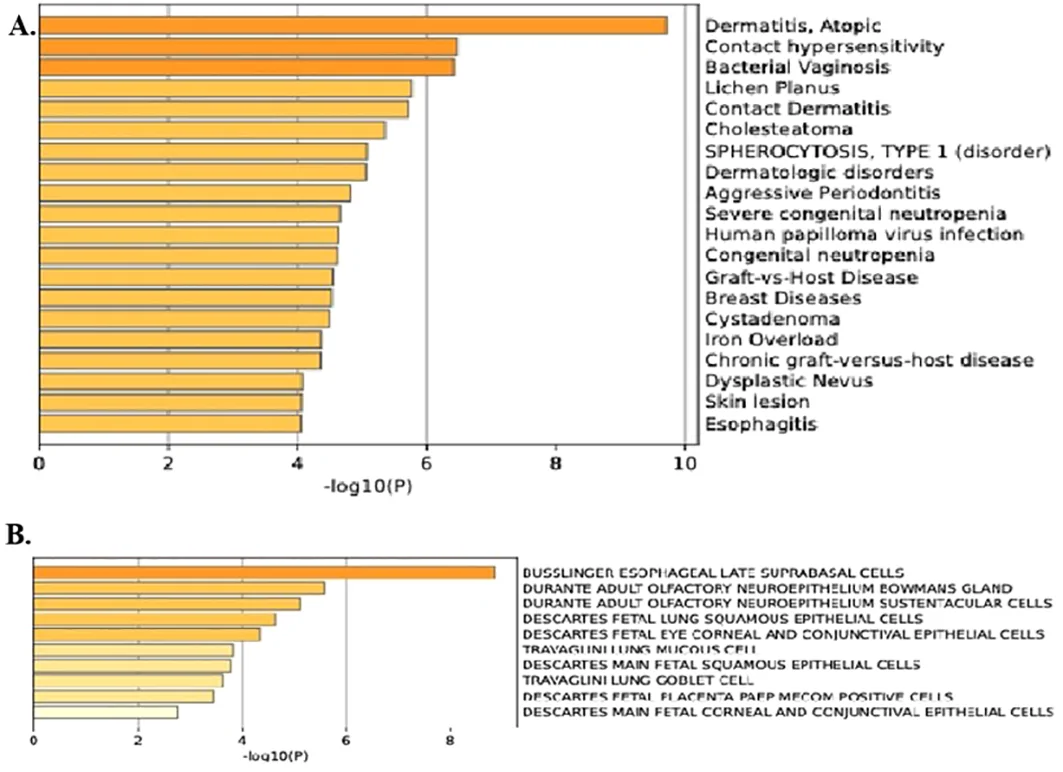
**(A)** Disease ontology and **(B)** cell-type enrichment analyses of common differentially expressed genes.

The GSE100681 and GSE58841 datasets yielded 19 commonly differentially expressed genes, with the majority showing upregulated patterns (**Figure 1**). The protein–protein interaction network established from the common genes was highly interconnected, with TP53 designated as the dominant hub node (**Figure 2**). TP53 also had the greatest closeness and betweenness centralities, providing additional evidence for its importance in regulating connectivity and information flow in this interaction network (**Figures 3**, **4**). TP53 exhibits functional relationships with several key regulatory molecules associated with the mitogen-activated protein kinase (MAPK) signaling pathways, including MAPK11, MAPK12, and MAPK13, suggesting involvement in stress response and tumorigenesis regulation (**Figure 5**). The robustness of the evidence supporting TP53’s regulatory importance was confirmed by stringent filtering and multiple priority algorithms (**Figure 4**). Furthermore, enrichment analysis linked these genes with epithelial cell types and disease processes associated with both inflammation and cancer.

### 3.2 *In silico* molecular docking

Molecular docking was performed to assess the interaction potential of a subset of selected FDA-approved compounds, shortlisted from a larger compound library, against the p53 protein within the HPV16-associated degradation complex (PDB ID: 4XR8). Docking simulations were carried out using Chain C and Chain D of 4XR8, which correspond to the p53 components within this ternary complex (HPV16 E6/E6AP/p53), and are functionally relevant to E6-mediated p53 destabilization.

**Figure 6:**
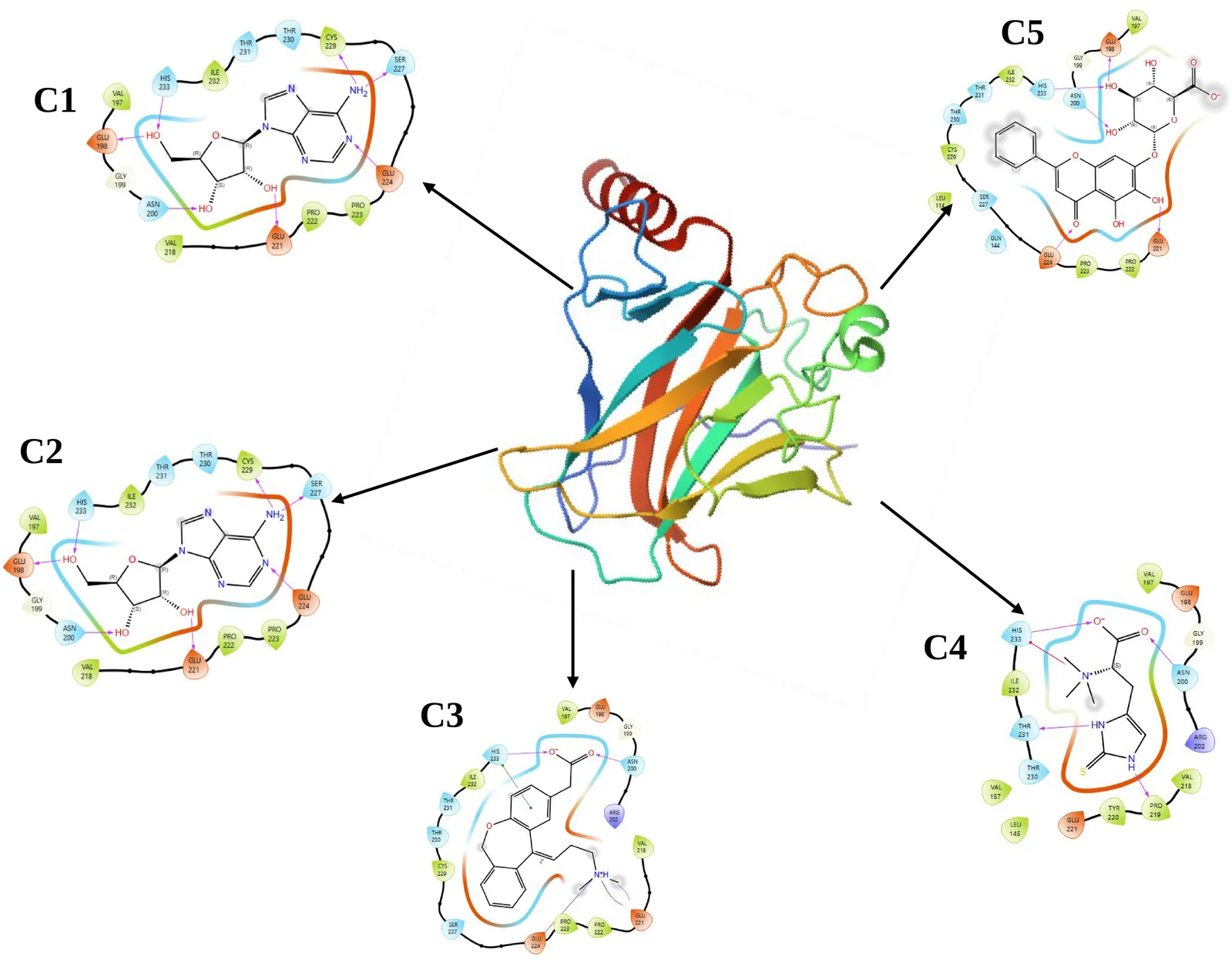
Molecular docking results of selected ligands with the target protein (PDB ID: 4XR8, Chain C). Three-dimensional ribbon representation of the 4XR8 (Chain C) protein structure, highlighting the ligand-binding region. (C1) Adenosine, (C2) Naloxone, (C3) Olopatadine, (C4) Ergothioneine, and (C5) Baicalin showing their respective binding conformations and key intermolecular interactions within the active site.

Adenosine (C1) had the highest binding affinity of −6.318 kcal/mol out of all compounds being evaluated and formed 7 hydrogen bonds. Naloxone (C2) was second with a binding affinity of −6.058 kcal/mol and 3 hydrogen bonds providing reasonable hydrogen-bonding potential. Similarly, olopatadine (C3) revealed a binding affinity of −6.041 kcal/mol and also formed 3 hydrogen bonds indicating moderately stable but relatively stable binding interactions in the binding pocket. Ergothioneine (C4) showed binding affinity of −5.864 kcal/mol and 4 hydrogen bonds whereas baicalin (C5) had a binding affinity of −5.780 kcal/mol and 5 hydrogen bonds producing important interactions with key active-site residues for the C chain as illustrated in **Figure 6**.

**Figure 7:**
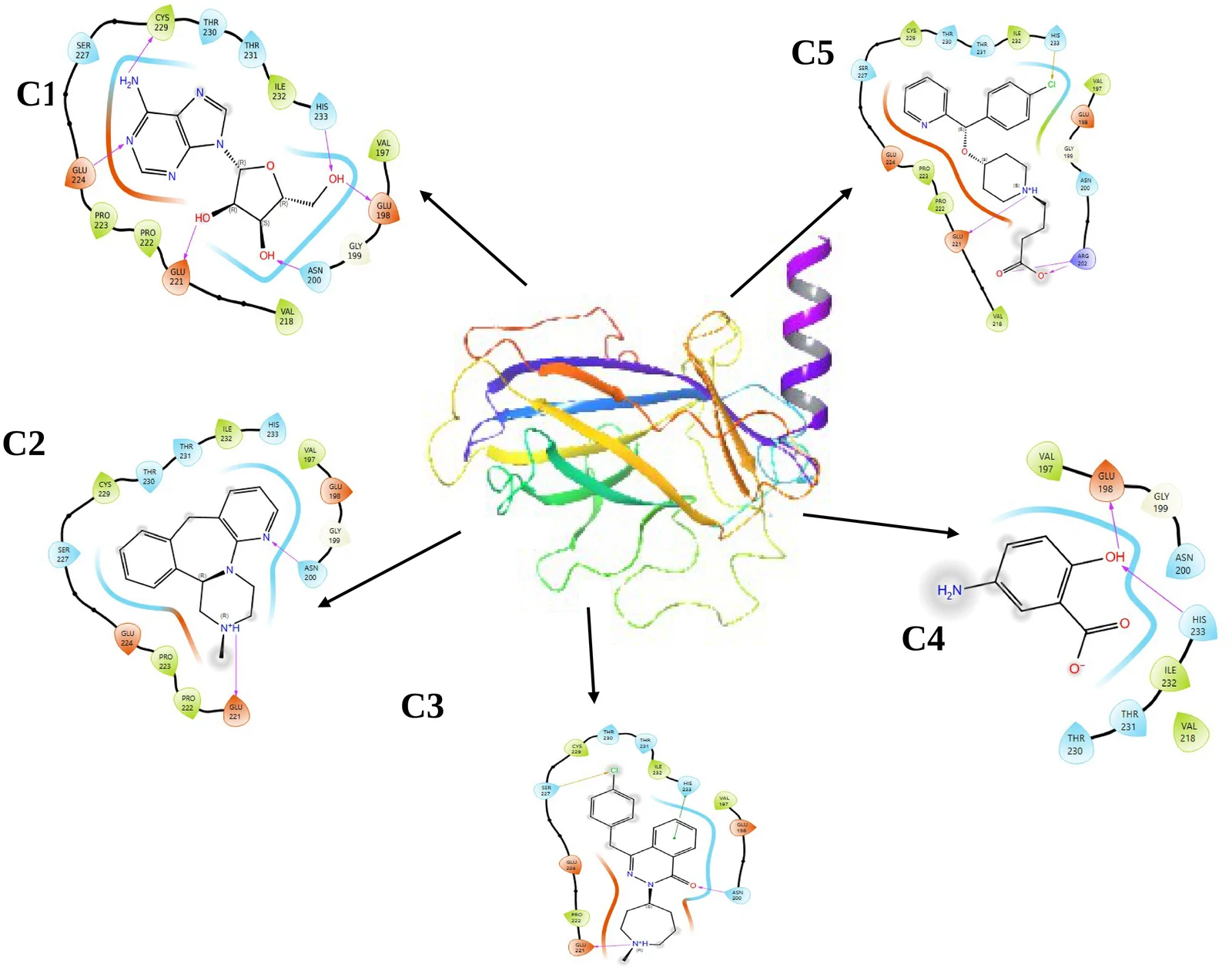
Molecular docking results of selected ligands with the target protein (PDB ID: 4XR8, Chain D). Three-dimensional ribbon representation of the 4XR8 (Chain D) protein, highlighting the ligand-binding pocket. (C1) Adenosine, (C2) Mirtazapine, (C3) Azelastine, (C4) Mesalamine, and (C5) Bepotastine showing their optimal docked conformations and key intermolecular interactions within the active site.

Adenosine (C1) bound strongly to Chain D, with six hydrogen bonds and a docking score of -7.104 kcal/mol. Mirtazapine (C2) maintained similar binding to Chain D with two hydrogen bonds giving it a stable interaction with a docking score of -5.923 kcal/mol kcal/mol. Azelastine (C3) had two hydrogen bonds and a docking score of -5.848 kcal/mol while Mesalamine (C4) also had two hydrogen bonds and a docking score of -5.636 kcal/mol. Bepotastine (C5) demonstrated very strong characteristics of interaction by maintaining three hydrogen bonds and exhibiting a docking score of -5.568 kcal/mol as shown in **Figure 7**.

The molecular docking studies demonstrates the ability of the FDA-approved drug adenosine to bind to chains C and D of HPV E6 with favorable binding interactions. In fact, because of the number of hydrogen bonds involved and longer duration of stable binding, adenosine exhibits the highest binding affinity when binding to either p53 chain within the 4XR8 complex. These results suggest that adenosine may occupy a region of the p53 interface relevant to E6 binding; however, whether this translates to competitive inhibition of E6-mediated p53 recruitment requires direct biochemical confirmation and subsequent proteasomal degradation. Collectively, these findings support adenosine as a candidate for further investigation as a possible lead compound for drug discovery.

### 3.3 DFT calculations

**Figure 8:**
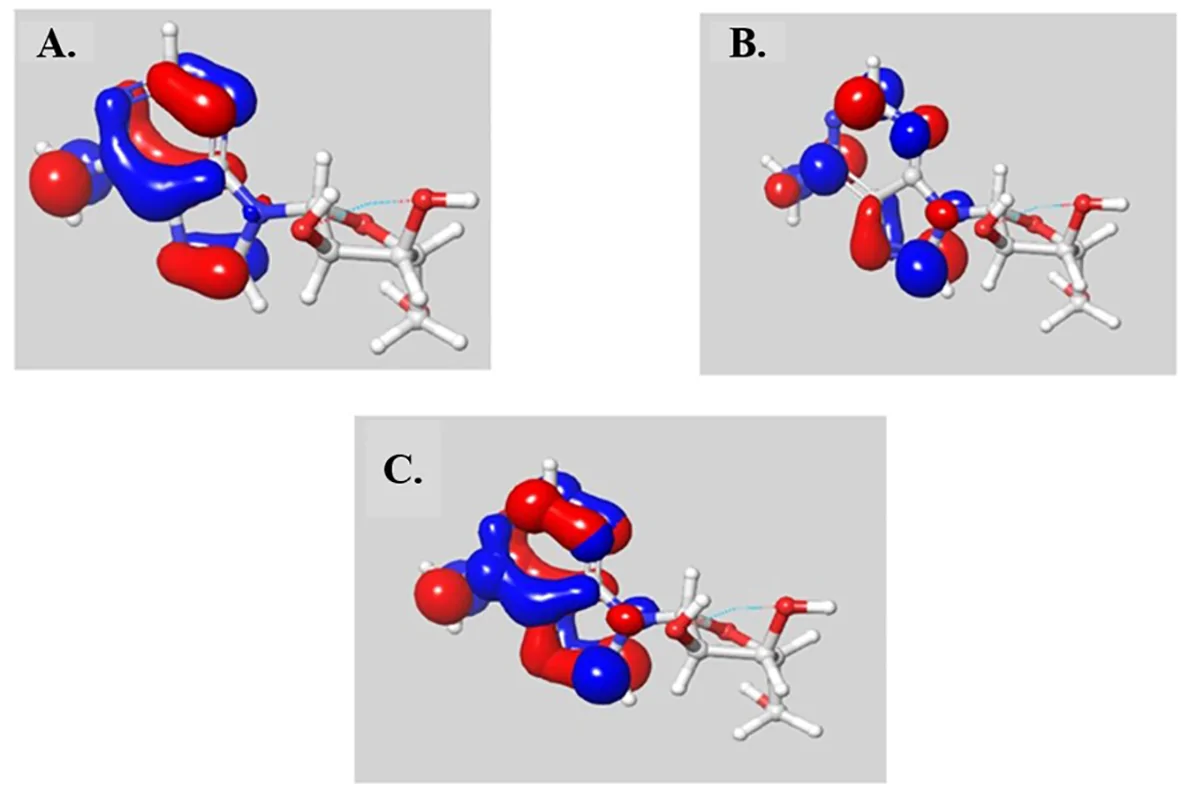
Frontier molecular orbital analysis depicting **(A)** HOMO, **(B)** LUMO, and **(C)** HOMO–LUMO overlap highlighting electron donor–acceptor regions relevant for molecular reactivity.

DFT analysis estimates how readily a molecule’s electrons can be donated or accepted, which provides a proxy for its chemical reactivity and stability when interacting with a protein-binding pocket; a larger HOMO-LUMO gap generally indicates a more electronically stable, less reactive molecule. The frontier molecular orbital parameters of adenosine were determined through DFT computational analysis to gain insight into its electronic characteristics relevant to protein binding. Specifically, the HOMO energy level was calculated at −0.215945 Hartree (−5.878 eV) and the LUMO energy level at −0.017504 Hartree (−0.476 eV). The frontier orbital energy gap (Egap) was 0.198441 Hartree, equivalent to 5.399 eV upon conversion (1 Hartree = 27.211 eV). This Egap value is consistent with moderate electronic reactivity of adenosine, supporting its capacity for protein–ligand interactions at the p53 binding interface (**Figure 8**).

### 3.4 ADMET study

ADMET profiling predicts, before any animal or human testing, whether a compound is likely to be adequately absorbed, distributed, metabolized and excreted in the body, and whether it carries obvious toxicity liabilities; this provides an early indication of drug-likeness and safety. Adenosine was subjected to in silico ADMET profiling via pkCSM, SwissADME, and ADMETlab3.0 to obtain complete information about its pharmacokinetics and toxicity. The results of the absorption analyses indicate that the compound has moderate aqueous solubility based on the three analysis platforms: pkCSM (−2.346 log mol/L), SwissADME (−1.39), and ADMETlab (−1.677), with an estimated intestinal absorption of 61% in humans. However, both SwissADME and ADMETlab classified the absorption probability as low. In addition, all of the platforms indicate that the compound meets Lipinski’s Rule of Five, indicating that the compound is likely a good candidate for drug development. There were variances in the predicted P-glycoprotein substrate classifications. SwissADME predicts potential substrate status, whereas pkCSM predicts no substrate status, with a moderate likelihood of being a substrate (ADMETlab: 0.390). A bioavailability score of 0.55 would indicate moderate oral bioavailability (**Table 1**).

**Table 1:** *In Silico* ADMET profile of adenosine predicted by pkCSM, SwissADME, and ADMETlab3.0.

Property class	Parameter	pkCSM prediction	SwissADME prediction	ADMETlab3.0 prediction	Standard unit/interpretation
Absorption	Water solubility	-2.346	-1.39	-1.677	log mol/L
	Human intestinal absorption	61.243	Low	Low	% absorbed
	Lipinski rule of five	Yes	Yes	Yes	Yes/No
	P-glycoprotein substrate	No	Yes	0.390	Yes/No
	Bioavailability score	-	0.55	-	Probability Distribution
Distribution	Volume of distribution (VDss)	0.844	-	–0.188	log L/kg
	BBB permeability	-1.23	No	0.653	log BB
Metabolism	CYP3A4 substrate	No	No	No	Yes/No
	CYP450 inhibition (1A2, 2C9, 2C19, 2D6, 3A4)	No	No	No	Yes/No
Excretion	Total clearance	0.763	-	-	log mL/min/kg
Toxicity	AMES mutagenicity	No	-	No	Yes/No
	hERG I inhibitor	No	-	No	Yes/No
	Oral rat acute toxicity (LD_50_)	1.864	-	-3.75	mol/kg
	Hepatotoxicity	No	-	No	Yes/No

Distribution parameters would suggest moderate volume of distribution (pkCSM: 0.844 log L/kg) and limited ability (pkCSM: −1.23 logBB) to crossing blood brain barrier, therefore reducing the ability to be exposed to the CNS. The metabolism analysis indicates no CYP3A4 liability and no inhibition of major CYP450 isoforms; thus, the risk of drug-drug interaction is low. The excretion analysis indicates satisfactory total clearance of the compound (0.763 log mL/min/kg). Toxicity predictions are consistent with the AMES negative result, absence of hERG I inhibition, no hepatotoxicity, and acceptable estimates of acute toxicity.

### 3.5 Molecular dynamics-based stability analysis of protein–ligand complex

The dynamic stability and conformational behavior of the D chain of 4XR8 complexed with adenosine and doxorubicin were analyzed through molecular dynamics simulations at 500ns using the Desmond module of the Schrödinger suite. Preliminary MD simulations were also conducted for the C chain complex; however, the C chain exhibited markedly greater conformational fluctuations compared to the D chain, with higher RMSD deviations throughout the trajectory, suggesting reduced complex stability. Consequently, the D chain was selected for comprehensive MD analysis and detailed interpretation. Dynamic stability of protein-ligand complexes was characterized through RMSD and RMSF calculations derived from molecular dynamics trajectories. The protein and ligand RMSD quantify the overall conformational stability over time, while protein RMSF characterizes residue-by-residue flexibility.

**Figure 9:**
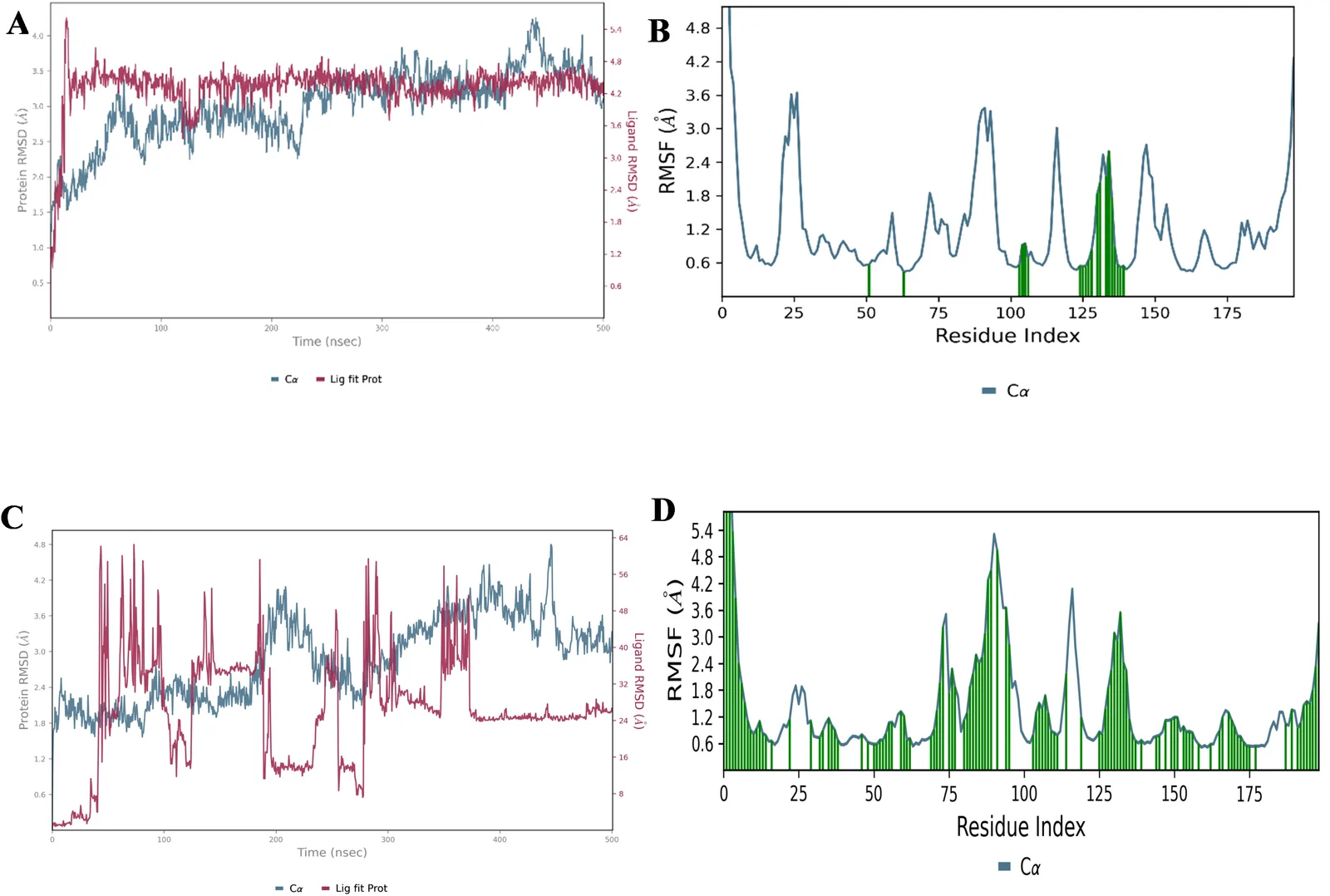
Molecular dynamics simulation analysis of 4XR8 (Chain D) complexes performed using Schrödinger–Desmond at 500 ns. **(A)** Time evolution of protein Cα RMSD and ligand RMSD for the adenosine-bound complex. **(B)** RMSF profile of chain D residues in the adenosine-bound system. **(C)** Protein and ligand RMSD for the doxorubicin-bound complex. **(D)** RMSF profile of chain D residues in the doxorubicin-bound system.

As shown in **Figure 9A**, after equilibration of the 4XR8-adenosine complex, the Cα RMSD of the protein remained relatively stable throughout, indicating that the global fold of chain D was maintained. The ligand RMSD, relative to the protein, showed only small fluctuations, indicating that adenosine remained stably anchored in the binding pocket and did not experience significant displacement. Correspondingly, the low fluctuations of the residue-level RMSF profile (**Figure 9B**) indicate that most residues displayed little flexibility, except a few flexible loop regions, while the residues that are in contact with the binding site displayed high rigidity, indicating a stable/excellent retention in the binding mode for adenosine.

When compared to the doxorubicin complex, the RMSD values show very large deviations during the early parts of the MD simulation of the 4XR8 – doxorubicin complex, followed by a stabilization of the RMSD values toward the end of the MD simulation. The doxorubicin – 4XR8 (protein-ligand) complex undergoes a maximum degree of conformational relaxation over the first few MD simulation time steps. The ligand RMSD (**Figure 9C**) exhibited spikes or periodic spikes throughout the MD simulation, indicating that doxorubicin undergoes periodic reorientation within the binding pocket and exhibits greater motion than other ligands. The RMSF analysis (**Figure 9D**) also supports this observation with chain D of 4XR8 exhibiting increased residue flexibility, particularly for residues located close to the binding site, indicating that doxorubicin binding induces considerable local conformational perturbations within the protein.

The overall observation from the MD simulation supports the conclusion that adenosine bound to the p53 D chain of 4XR8 (within the E6/E6AP/p53 ternary complex) with greater stability than the comparator doxorubicin, as evidenced by lower RMSD and RMSF values, based on the evaluation of the RMSD and RMSF deviations calculated from the simulation data. The dynamic stability profiles are consistent with the docking studies and reinforce that the test ligand has desirable binding behaviors under simulated physiological conditions. As with the docking analysis, these MD findings demonstrate conformational stability of the predicted complex rather than functional inhibition of E6-mediated p53 degradation, which remains to be established biochemically.

### 3.6 Free radical scavenging activity using DPPH

**Figure 10:**
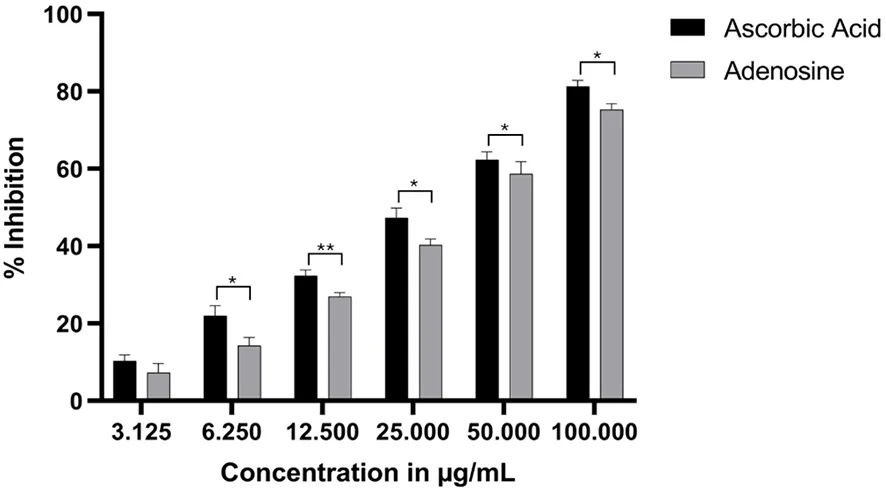
Dose-dependent percentage inhibition of ascorbic acid and adenosine at varying concentrations (3.125–100 µg/mL). Data expressed as mean ± SD (n = 3). IC50: ascorbic acid = 29.50 µg/mL; adenosine = 38.20 µg/mL. Statistical significance: *p < 0.05, **p < 0.01, ***p < 0.001 (two-way ANOVA, Tukey *post-hoc* test).

Adenosine’s antioxidant potential was compared to that of ascorbic acid (standard antioxidant) at different concentrations (3.125-100 μg/mL). Ascorbic acid had strong activity with an IC_50_ of 29.50 μg/mL, and Adenosine had a marginally higher IC_50_ of 38.20 μg/mL, indicating that ascorbic acid is marginally more potent as a free-radical scavenger. Nevertheless, adenosine showed comparable antioxidant activity, with both compounds achieving approximately 80% inhibition at 100 µg/mL, confirming a dose-dependent free-radical scavenging response (**Figure 10**).

### 3.7 FRAP-based antioxidant activity assessment

**Figure 11:**
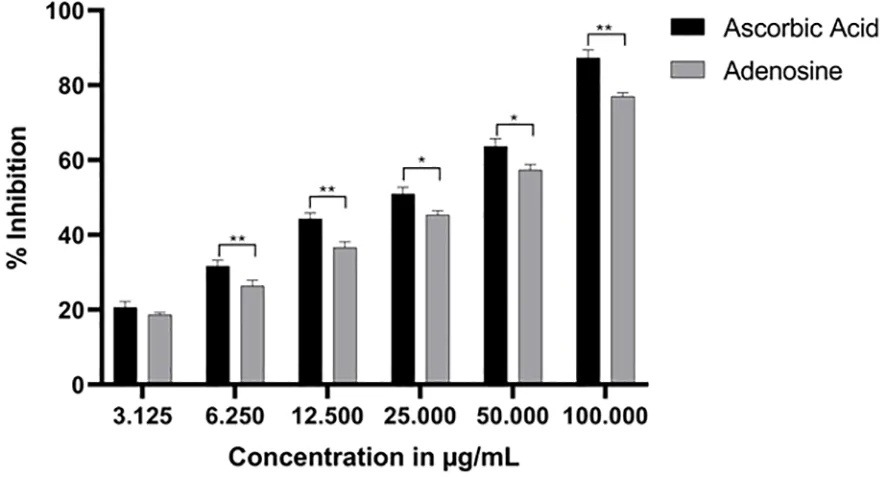
Ferric Reducing Antioxidant Power (FRAP) assay showing concentration-dependent % inhibition of ascorbic acid and adenosine (3.125–100 µg/mL). Data expressed as mean ± SD (n = 3). IC50: ascorbic acid = 23.10 µg/mL; adenosine = 34.80 µg/mL. Statistical significance: *p < 0.05, **p < 0.01, ***p < 0.001 (two-way ANOVA, Tukey *post-hoc* test).

Antioxidant potential of Adenosine was assessed using the FRAP assay, with ascorbic acid serving as the positive control standard. Generally, both compounds showed consistently significant concentration-dependent increases in reducing activity against Fe^3+^ across the whole range of concentrations (3.125–100 μg/mL). Ascorbic acid had very strong reducing power with an IC_50_ of 23.10 μg/mL, which is consistent with the highly regarded electron-donating and antioxidant property of ascorbic acid. Adenosine did not show strong reducing activity as ascorbic acid, but it did show moderate reducing ability with an IC_50_ of 34.80 μg/mL. Adenosine’s activity consistently progressed in accordance with increasing concentration, but was reduced at all concentrations in comparison to ascorbic acid, which was a prominent finding. This ferric reducing effect may be attributable to the structure of Adenosine, allowing very little electron transfer to occur to reduce Fe^3+^ to Fe²^+^ (**Figure 11**).

### 3.8 Anti-inflammatory assay

**Figure 12:**
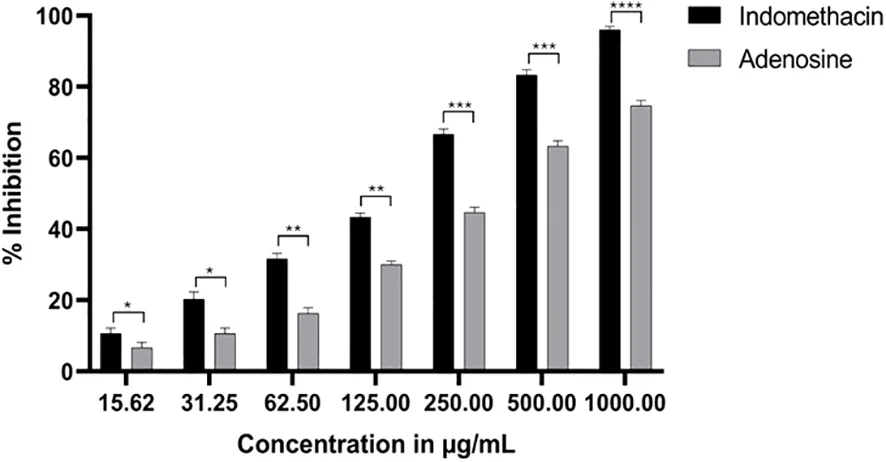
Concentration-dependent lipoxygenase inhibitory activity (% inhibition) of adenosine and indomethacin at concentrations ranging from 15.62 to 1000 µg/mL. Indomethacin (IC50 = 163.10 µg/mL) demonstrated markedly greater potency compared to adenosine (IC50 = 321.20 µg/mL). Data expressed as mean ± SD (n = 3). Statistical significance: **p < 0.01, ***p < 0.001, ****p < 0.0001 (two-way ANOVA, Tukey *post-hoc* test).

The anti-inflammatory activity of Adenosine was evaluated in comparison with indomethacin, a standard non-steroidal anti-inflammatory drug, over a concentration range of 15.62–1000 μg/mL. Both compounds studied inhibited inflammatory signaling in a concentration dependent manner, with indomethacin producing far greater inhibitory potencies than adenosine (**Figure 12**). At 163.10 μg/mL indomethacin produced 50% inhibition. In contrast, adenosine produced greater than 70% inhibition at the highest concentration tested but overall was shown to have less potency than indomethacin based on an IC50 of 321.20 μg/mL and produced only gradual and concentrations dependent increases in percentage inhibition until reaching the maximum concentration tested. Across the entire concentration range, inhibition by adenosine remained consistently lower than that of indomethacin, suggesting that Adenosine’s anti-inflammatory effect is moderate relative to the reference drug.

### 3.9 MTT assay

**Figure 13:**
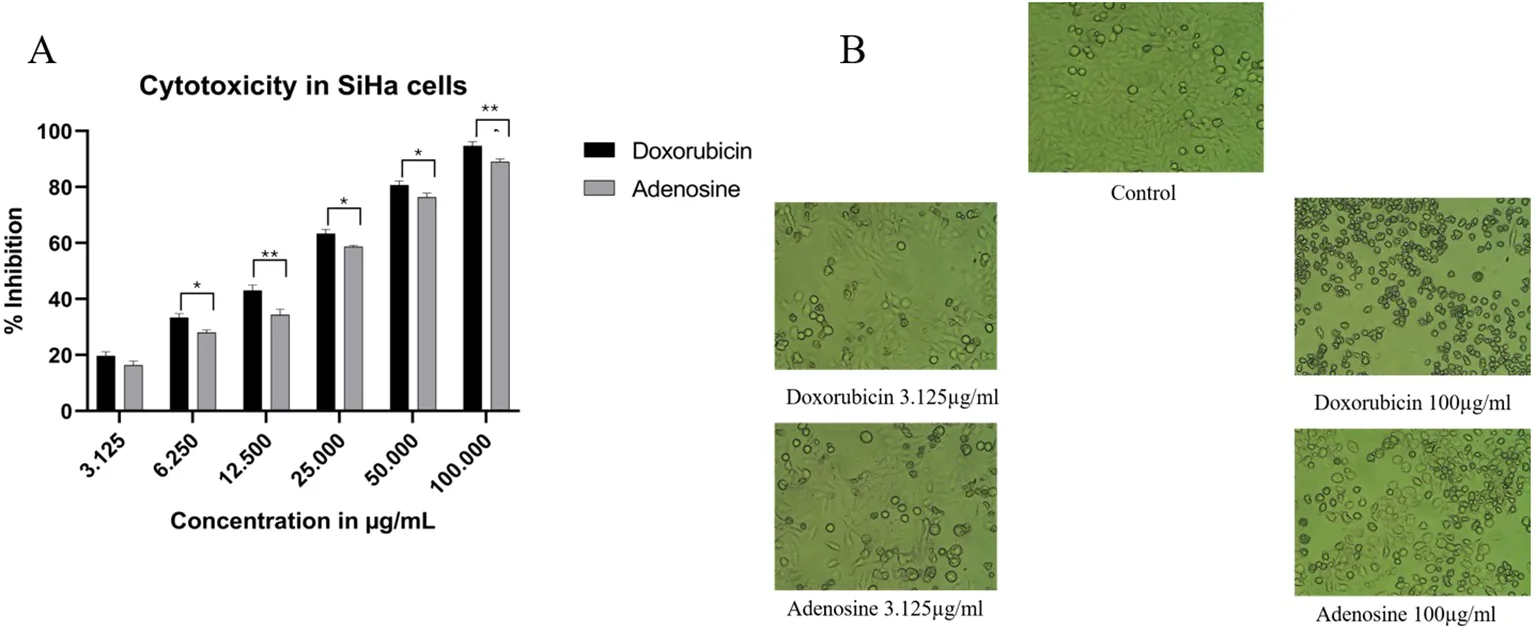
**(A)** Cytotoxic effects of adenosine and doxorubicin on SiHa cervical cancer cells determined by the MTT assay. Dose–response curves demonstrate concentration-dependent inhibition of cell viability following treatment with concentrations ranging from 3.125–100 µg/mL. Doxorubicin exhibited an IC_50_ value of 16.8 µg/mL, while adenosine showed an IC_50_ value of 20.6 µg/mL. Data are presented as mean ± SD from three independent experiments (n = 3). Statistical significance was determined by one-way ANOVA followed by Tukey’s *post hoc* test (p < 0.05, p < 0.01, p < 0.001). **(B)** Representative bright-field micrographs of SiHa cells following treatment with adenosine and doxorubicin, demonstrating similar concentration-dependent morphological changes characterized by reduced cell density, cell rounding, shrinkage, loss of adhesion, and cellular detachment, with greater morphological disruption in doxorubicin-treated cells than in adenosine-treated cells.

The effects of adenosine on the growth of SiHa cell lines were measured in an MTT assay for the determination of cell growth and compared to doxorubicin over a concentration range of 3.125-100 µg/mL. Both agents resulted in a significant reduction in cell viability at all concentrations when administered in a dose-dependent manner. All experiments were performed in triplicate (n=3) and data are expressed as mean ± SD. Statistical significance was determined by one-way ANOVA with Tukey *post-hoc* test (p < 0.05). Doxorubicin was determined to be much more effective with an IC_50_ of 16.8 µg/mL in SiHa cells as compared to adenosine’s IC_50_ of 20.6 µg/mL in SiHa cells, demonstrating that adenosine has very strong antiproliferative activity in SiHa cell (**Figure 13A**).

Doxorubicin produced a marked reduction in cell viability across the tested concentration range (3.125–100 µg/mL), with near-complete growth inhibition observed at the highest concentration tested (100 µg/mL). Adenosine likewise exhibited a concentration-dependent antiproliferative effect, SiHa cells adenosine’s activity approached that of doxorubicin, reflecting the closer IC_50_ values obtained in this HPV16-positive line. Morphological examination of SiHa cells following treatment revealed characteristic features of cytotoxicity, including reduced cell density, cell rounding, shrinkage, and loss of cell–substrate adhesion. These morphological alterations were more pronounced in doxorubicin-treated cells than in adenosine-treated cells, consistent with the lower IC_50_ values observed for doxorubicin (16.8 µg/mL in SiHa) compared with adenosine (20.6 µg/mL in SiHa) (**Figure 13B**). The MTT assay results demonstrated that adenosine significantly inhibited the proliferation of both cervical cancer cell lines in a dose-dependent manner, showing strong antiproliferative activity in SiHa cells to the standard chemotherapeutic agent doxorubicin. These findings are in agreement with the computational predictions and further support a genotype-independent antiproliferative effect for adenosine across both HPV16-positive, reinforcing its potential as a promising lead for cervical cancer. Nevertheless, further mechanistic investigations and *in vivo* studies are required to elucidate its molecular mode of action and validate its therapeutic efficacy.

### 3.10 CAM assay

**Figure 14:**
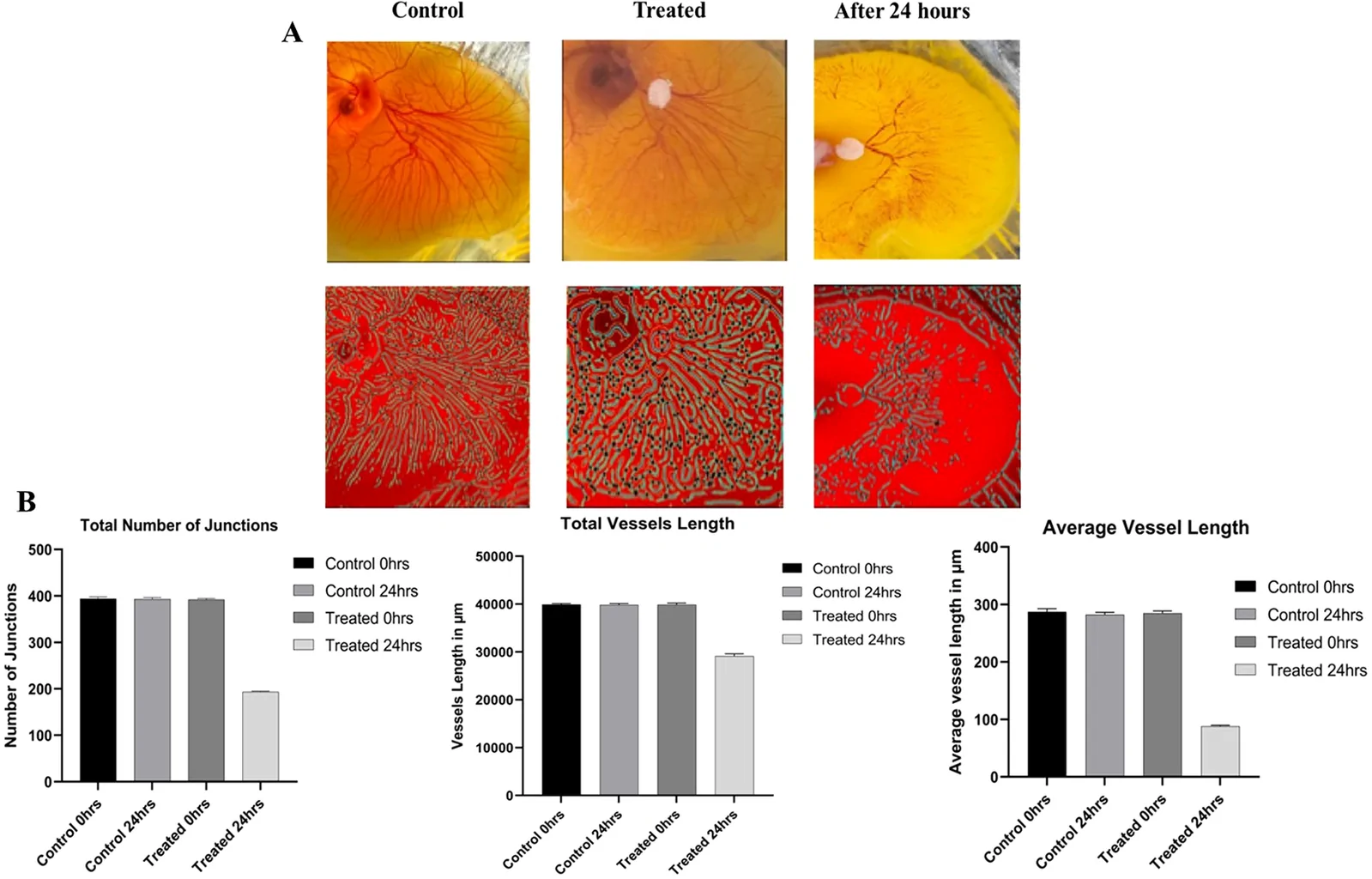
Anti-angiogenic effect of adenosine on the chick CAM assay. **(A)** Representative stereomicroscopic images of CAMs from control (untreated), adenosine-treated (0 hrs), and adenosine-treated (24 hrs) groups (top panel), with corresponding AngioTool-generated skeletonised vessel segmentation overlays (bottom panel). **(B)** Bar graphs depicting AngioTool-quantified vascular parameters: total number of junctions, total vessel length (µm), and average vessel length (µm) across all four groups (n = 3 per group). Data are presented as mean ± SD.

Adenosine’s anti-angiogenic properties were assessed using a chick chorioallantoic membrane assay. The vascularity of the membrane was measured using the AngioTool image analysis program to quantify various angiogenic parameters related to blood vessels (angiogenesis) (**Figure 14A**). The initial morphological evaluation showed a greatly enhanced vascularity for the control group compared to the adenosine-treated group. For example, the adenosine-treated group had significantly reduced vascular density as well as an apparent area of low or absent vascularity directly at the site of application of the compound. Vascular regression was even more pronounced by 24 hours following treatment, with significantly reduced vascular density compared with both the baseline controls and the early 0-hour controls. Quantitative analysis with AngioTool comparing three vascular parameters (total number of junctions, average vessel length, total vessel length) between all four summary groups showed no significant differences between the Control 0-hour, Control 24 hour, or Adenosine 0-hour groups, demonstrating that the morphology that was measured at baseline was stable and that vascularity of the two treatment groups was similar at the time of the first treatment. Following 24 hours of adenosine treatment, however, all three parameters were substantially reduced. The total number of junctions declined by approximately 50.7%, reflecting significant disruption of vascular branching and network complexity. Average vessel length decreased dramatically by approximately 69%, consistent with potent inhibition of angiogenic vessel elongation. There was a sizeable decrease in total vessel length of approximately 26.9%, further confirming that vascular networks are overall inhibited in development (**Figure 14B**). Both mean section length and junction totals are greatly decreased as compared to total vessel length, indicating that adenosine mainly interferes with branching and elongation of vessels rather than simply decreasing gross density of vessels. These findings highlight the significant and time-dependent anti-angiogenic activity of adenosine on the CAM model, indicating that adenosine exhibits potent anti-angiogenic activity warranting further investigation.

treated (0 hrs.), and adenosine-treated (24 hrs.) groups (top panel), with corresponding AngioTool-generated skeletonized vessel segmentation overlays (bottom panel), illustrating progressive vascular regression and reduction in network complexity following adenosine treatment. (B) Bar graphs depicting AngioTool-quantified vascular parameters: total number of junctions, total vessel length (µm), and average vessel length (µm) across Control 0 hrs., Control 24 hrs., Adenosine-Treated 0 hrs., and Adenosine-Treated 24 hrs. groups (n = 3 per group). Data are presented as mean ± SD.

## 4 Discussion

High-risk HPV persistent infection drives cervical carcinogenesis via E6 oncoprotein-induced p53 degradation (8, 9). When the p53 protein is degraded, it leads to impairment of apoptosis, defective DNA repair mechanisms, and genomic instability (10, 12). Currently, there are advances in both vaccination and immunotherapy (15). However, there is no clinical agent specifically targeting the E6 region of HPV (11, 19). In this study, we applied an integrative computational-experimental drug repurposing approach to identify compounds that may block HPV16 E6 and assessed their biological relevance.

Network pharmacology analyses of HPV E6/E7-expressing keratinocyte datasets identified TP53 as the primary molecular hub within the disrupted interaction network. While this finding is consistent with the well-established role of E6 in p53 degradation, it should be noted that the identification of TP53 as a hub gene from these datasets does not independently validate adenosine as a p53-stabilising or E6-targeting compound. Rather, it provides biological rationale for prioritizing the p53 interface as a docking target, which was subsequently pursued through molecular docking against the HPV16 E6/E6AP/p53 ternary complex (PDB: 4XR8) for treating HPV-induced cervical cancer.

Computationally, protein–ligand molecular docking identified adenosine as the most promising candidate based on its favorable binding affinities to both p53 chains (C and D) of the HPV16/E6AP/p53 ternary complex (4XR8). These interactions at the p53 interface may confer a stabilizing effect against E6-mediated degradation. The presence of hydrogen bonds, along with consistent docking scores, supports a stable interaction. The DFT calculations supported that the HOMO–LUMO energy gap of adenosine indicates sufficient electronic stability to support protein–ligand interactions (32).

The molecular dynamics simulations furthermore provide dynamic validation of the previous docking results. The adenosine–E6, D chain complex demonstrated stable RMSD and very little RMSF variation when compared to doxorubicin, indicating stable binding of adenosine to the HPV16 E6 protein, with only minor conformational deviations. Unlike adenosine, doxorubicin demonstrated structural differences from the uncomplexed form and underwent ligand reorientation, indicative of a less stable interaction with E6 under simulated physiological conditions.

Using an in silico ADMET assessment, we found that predicted pharmacokinetics include at least moderate solubility, good intestinal absorption, Lipinski’s pharmacokinetic rule compliance, little potential to inhibit CYP 450 enzymes, and no significant cardiac toxicity was predicted in silico. In addition, there was variability in predicting P-glycoprotein substrates; however, the overall pharmacokinetic profile is consistent with drug-likeness criteria for oral administration (33–35). The predicted low permeability through the blood-brain barrier may result in less central nervous system toxicity.

These computational predictions were supported by direct experimental *in vitro* data. Adenosine exhibited concentration-dependent cytotoxicity in SiHa cells, with lower potency than doxorubicin in both cell lines overall; however, its antiproliferative activity was comparatively stronger in the HPV16-positive SiHa cell line (IC_50_ = 20.6 µg/mL), approaching that of doxorubicin (IC_50_ = 16.8 µg/mL) in this line. Additionally, it interacted positively in multiple antioxidant assays (DPPH and FRAP) (37, 38) and exhibited moderate anti-inflammatory activity via lipoxygenase inhibition (40), and was very effective in inhibiting angiogenic proliferation in the chicken embryo CAM model system of angiogenesis (42, 43). Together, these multiple properties can provide potential additive therapeutic effects in treating cervical cancer. Placed alongside previously reported repurposing efforts against the HPV E6/p53 axis, including the docking-based identification of valganciclovir and cytarabine as candidate E6-binding agents (19). Adenosine’s cytotoxic approaches a more potent range in the HPV16-positive SiHa cell line (IC_50_ = 20.6 µg/mL), where it is only modestly less active than doxorubicin (IC_50_ = 16.8 µg/mL) under the same assay conditions. This genotype-dependent difference in potency is itself noteworthy, and may reflect underlying differences in adenosine receptor expression, nucleoside transporter activity, or baseline p53 pathway integrity that warrants further investigation.

Although adenosine has been studied extensively for its cardiovascular and immune-modulatory properties and is produced in the human body (44–46), little is known about its use in inhibiting HPV E6 target molecular pathways. Our findings indicate that the repurposing of adenosine may provide a new approach to blocking E6-mediated suppression of p53 function. Several limitations must be acknowledged. First, the docking and MD simulations were performed against p53 chains (Chains C and D) of the HPV16 E6/E6AP/p53 ternary complex (PDB: 4XR8); direct E6 binding assays or E6-targeted docking using isolated E6 chains were not performed in this study and represent a priority for future work. Second, no direct biochemical assays (e.g., co-immunoprecipitation, p53 ubiquitination assays, Western blotting of p53 levels post-treatment) were conducted to validate p53 restoration or E6 inhibition.

Despite the favorable computational and preliminary *in vitro* profile of adenosine, several important translational limitations must be explicitly acknowledged. Adenosine that is produced within the body has a very short amount of time in the bloodstream to work (~10 seconds) because the cells in the body take up adenosine through a nucleoside transporter and then break it down through the action of an adenosine-deaminase enzyme. These actions effectively make the use of adenosine as an oral or injectable medicine very ineffective because of how little adenosine there would actually be available in the blood for therapeutic use (45, 46). Adenosine also has multiple conflicting effects on the body’s immune system in relation to cancer, wherein stimulation via adenosine receptor stimulation (A2A and A2B primarily) appears to suppress or modify anti-cancer immune response (47, 48); yet, the elevation of adenosine level near the tumor could stimulate an immune response either to eliminate the tumor or to participate in the anti-cancer immune response (46, 49). Due to the complexity of the pharmacology associated with adenosine, it would seem improbable that any current route of delivery will be clinically viable without formulating such that significant advances towards encapsulation in nanoparticles, developing pro-drugs, or employing specialized delivery methods have occurred (50). Several concrete strategies could be explored in follow-up work to overcome this barrier: nanoparticle or liposomal encapsulation to shield adenosine from rapid deaminase-mediated breakdown and enable tumor-localized release; stable prodrug derivatives designed to resist adenosine deaminase and release active adenosine slowly at the target site; and localized or intratumoral delivery approaches that would limit systemic exposure and the associated immune-modulatory effects mediated by A2A/A2B receptors.

The *in vitro* findings regarding adenosine should also be investigated through use of the known receptors (A1, A2A, A2B, A3R) that are found in cervical cancer cells; if not, future studies should use ectopic expression of the above-mentioned types of cells with either receptor-specific blockers or genetic deletion of p53 in cell lines to separate out the potential pathways of the biological effects caused by the adenosine receptor signaling systems (51). Inflammation, angiogenesis and proliferation of cells can all be influenced by ways other than just direct p53 binding (as predicted from calculations). Thus, future studies will most likely require the utilization of receptor specific inhibitors and p53 deletion in the investigation of these processes. Future studies should therefore focus not only on mechanistic validation but also on strategies to overcome these pharmacokinetic barriers before adenosine can be considered a translatable therapeutic candidate.

Overall, the results of this study provide mechanistic, computational, and preliminary experimental evidence to support continued investigation into adenosine’s potential use as an inhibitor of HPV E6. Additional studies will be required to establish the therapeutic potential for cervical cancer through further mechanistic validation, optimization, and translational investigations.

## 5 Conclusion

This integrated computational and preliminary *in vitro* study identifies adenosine as a promising candidate warranting further investigation as a modulator of the HPV E6–p53 interaction in cervical cancer, based on results obtained using a number of analyses such as Integrated Network Pharmacology, Molecular Docking, Density Functional Theory (DFT), ADMET and Molecular Dynamics Studies. Adenosine showed the most favorable predicted binding, among the compounds screened, against both Chain C and Chain D of the HPV16 E6/E6AP/p53 complex (PDB: 4XR8). Chain D yielding the highest docking score of -7.104 kcal/mol which is vigorously stable over 500ns in an MD Study as compared to doxorubicin.

Adenosine also meet drug-likeness criteria, good pharmacokinetic properties, and electronically adenosine can be classified as a potential candidate for a lead drug. The preliminary studies showed a concentration dependent anti-cancer effect using SiHa cells (IC_50_ = 20.6 µg/mL). Antioxidant, moderate anti-inflammatory, and notable anti-angiogenic effects determined using the CAM model further support the findings.

The above findings highlight a potential link between adenosine and the p53 interface, which may lead to new methods of treating HPV-associated cervical cancer. The possibility exists for adenosine to counteract the E6-mediated degradation of p53 through its interaction with this interface. Because of these reasons, the authors feel that further studies focused on p53 as a new therapeutic target provide a basis for future studies. However, several limitations should be noted. No direct biochemical data support the ability of adenosine to stabilise p53; only *in vitro* data using HPV16-positive SiHa cells were obtained for this study. Addressing the limitation of adenosine through nanoparticle, prodrug, or localized-delivery strategies remains necessary before adenosine can be considered a viable therapeutic agent. Prior to considering adenosine as a viable therapeutic agent for HPV-associated cervical cancer, direct biochemical assays are needed to evaluate the ability of p53 to be restored by adenosine. In addition, studies using receptor-specific blockers may help to distinguish the role of p53 in adenosine action from the role of adenosine receptors. Finally, additional studies may be necessary for evaluating *in vivo* efficacy before adenosine may be considered a therapeutic agent.

## Data Availability

The datasets presented in this study can be found in online repositories. The names of the repository/repositories and accession number(s) can be found below: https://www.ncbi.nlm.nih.gov/geo/, GSE100681 https://www.ncbi.nlm.nih.gov/geo/, GSE58841.
